# Evaluation of the Organotellurium Compound AS101 for Treating Colistin- and Carbapenem-Resistant *Klebsiella pneumoniae*

**DOI:** 10.3390/ph14080795

**Published:** 2021-08-12

**Authors:** Tsung-Ying Yang, Hao-Yun Kao, Po-Liang Lu, Pei-Yu Chen, Shu-Chi Wang, Liang-Chun Wang, Ya-Ju Hsieh, Sung-Pin Tseng

**Affiliations:** 1 Department of Medical Laboratory Science and Biotechnology, College of Health Sciences, Kaohsiung Medical University, Kaohsiung 807, Taiwan; zegma040899@gmail.com (T.-Y.Y.); specialforevery8@gmail.com (P.-Y.C.); shuchiwang@kmu.edu.tw (S.-C.W.); 2 Department of Healthcare Administration and Medical Informatics, Kaohsiung Medical University, Kaohsiung 807, Taiwan; haoyun@kmu.edu.tw; 3 Center for Liquid Biopsy and Cohort Research, Kaohsiung Medical University, Kaohsiung 807, Taiwan; d830166@gmail.com; 4 Division of Infectious Diseases, Department of Internal Medicine, Kaohsiung Medical University Hospital, Kaohsiung 807, Taiwan; 5 School of Post-Baccalaureate Medicine, College of Medicine, Kaohsiung Medical University, Kaohsiung 807, Taiwan; 6 Center for Cancer Research, Kaohsiung Medical University, Kaohsiung 807, Taiwan; 7 Department of Marine Biotechnology and Resources, National Sun Yat-sen University, Kaohsiung 80424, Taiwan; marknjoy@g-mail.nsysu.edu.tw; 8 Department of Medical Imaging and Radiological Sciences, Kaohsiung Medical University, Kaohsiung 807, Taiwan; 9 Drug Development and Value Creation Research Center, Kaohsiung Medical University, Kaohsiung 807, Taiwan; 10 Department of Medical Research, Kaohsiung Medical University Hospital, Kaohsiung 807, Taiwan; 11 Graduate Institute of Animal Vaccine Technology, College of Veterinary Medicine, National Pingtung University of Science and Technology, Pingtung 900391, Taiwan

**Keywords:** carbapenem resistance, colistin resistance, *Klebsiella pneumoniae*, sepsis mouse model, AS101, drug repurposing

## Abstract

Colistin- and carbapenem-resistant *Enterobacteriaceae* cases are increasing at alarming rates worldwide. Drug repurposing is receiving greater attention as an alternative approach in light of economic and technical barriers in antibiotics research. The immunomodulation agent ammonium trichloro(dioxoethylene-*O*,*O*’-)tellurate (AS101) was repurposed as an antimicrobial agent against colistin- and carbapenem-resistant *Klebsiella pneumoniae* (CRKP). 134 CRKP isolates were collected between 2012 and 2015 in Taiwan. The in vitro antibacterial activities of AS101 was observed through broth microdilution, time-kill assay, and electron microscopy. Pharmaceutical manipulation and RNA microarray were applied to investigate these antimicrobial mechanisms. *Caenorhabditis elegans*, a nematode animal model, and the Institute for Cancer Research (ICR) mouse model was employed for the evaluation of in vivo efficacy. The in vitro antibacterial results were found for AS101 against colistin- and CRKP isolates, with minimum inhibitory concentration (MIC) values ranging from <0.5 to 32 μg/mL. ROS-mediated antibacterial activity eliminated 99.9% of bacteria within 2–4 h. AS101 also extended the median survival time in a *C. elegans* animal model infected with a colistin-resistant CRKP isolate and rescued lethally infected animals in a separate mouse model of mono-bacterial sepsis by eliminating bacterial organ loads. These findings support the use of AS101 as an antimicrobial agent for addressing the colistin and carbapenem resistance crisis.

## 1. Introduction

*Klebsiella pneumoniae* is a well-studied, Gram-negative pathogen associated with serious nosocomial and community-acquired infections [1]. Standard *K. pneumoniae* infection treatments include β-lactams, such as cephalosporins [2]. The extended-spectrum β-lactamase (ESBL)-producing isolates that were resistant to cephalosporins would be treated with carbapenems [3]. Carbapenem resistance has increased worldwide due to its expanded usage [4,5], thereby limiting therapeutic options for *K. pneumoniae* infections and encouraging the re-introduction of colistin as a last resort [6]. However, increased colistin consumption has triggered colistin-resistance rates as high as 53% in Zimbabwe, 11–52% throughout Europe (especially Greece), and 14–50% in Asia [7,8,9].

Epidemiological data indicate an urgent need to develop new antibiotics or alternative treatment methods for colistin- and carbapenem-resistant *K. pneumoniae* (CRKP). However, antibiotic development requires long lead times and large resource investments [10,11], therefore, researchers are looking at drug repurposing to meet the clinical treatment requirements of drug-resistant bacterial infections [12]. The attractiveness of drug repurposing is tempered by evidence showing that effective concentrations against Gram-negative bacteria exceed the maximum human plasma concentrations for drugs such as bleomycin and auranofin, among others [13]. Of 1040 Food and Drug Administration (FDA)-approved drugs tested, only five expressed bacteriostatic activity against MDR *Acinetobacter baumannii* ATCC BAA-1605 [14]. In light of the multiple challenges that repurposed FDA-approved drugs aimed at antibiotic-resistant Gram-negative bacteria must overcome, our screening strategy included some current clinical trial drugs and small compound libraries for activity-based drug discovery [15]. One low molecular weight organo-tellurium compound, ammonium trichloro(dioxoethylene-*O*,*O*’-)tellurate (AS101), expressed unexpected antimicrobial activity against the colistin-resistant CRKP isolate CRE-723. In the late 1980s, AS101 was used as an immunomodulation agent for cytokine secretions such as IL-1α, TNFα, and IL-2 [16,17]. Toxicologists have reported a 50% cytotoxic concentration (CC_50_) of AS101 in Vero cells of 145 μg/mL, with no cell damage observed at concentrations below 100 μg/mL, and with a 5–10 mg/kg 50% lethal dose (LD_50_) for intravenous injections in mice [18,19]. According to the ClinicalTrials.gov website, several phase II clinical trials involving AS101 as an immunomodulation agent alone or in combination with other medications are currently in progress or recently completed. Encouraged by these efforts, we evaluated AS101 as a repurposed “lifesaver” against colistin-resistant CRKP isolates by studying its in vitro activities, antibacterial mechanisms, in vivo effectiveness in a *Caenorhabditis elegans* infection model, and treatment efficacies in a mouse model of mono-bacterial sepsis.

## 2. Results

### 2.1. In Vitro AS101 Antibacterial Activity

Among the 134 CRKP isolates we examined, high resistance rates (95.6–100%) were determined in 15 antibiotics: aztreonam, piperacillin-tazobactam, cefazolin, cefoxitin, ceftazidime, cefotaxime, ceftriaxone, cefepime, imipenem, meropenem, ertapenem, doripenem, ciprofloxacin, levofloxacin, and trimethoprim/sulfamethoxazole. Moderate resistance rates were observed in gentamicin (86/134, 64.2%), colistin (55/134, 41.0%) and amikacin (43/134, 32.1%) (Figure S1). We identified 107 isolates (79.9%) with ESBL genes, 60 with AmpC (44.8%), and 66 with carbapenemases (49.3%) (Figure S2). Results from an epidemiological investigation indicate that among 27 pulsotypes, type I was the predominant clone (46.3%, 62/134) and ST-11 was the most frequent sequence type (78.4%, 105/134) (Figure S3). An ST-11, colistin-resistant, and KPC-producing isolate CRE-723 was selected for further assessments.

AS101 antibacterial activity involving the 134 CRKP isolates was investigated in vitro. As shown in Table 1, MIC values for colistin-susceptible and colistin-resistant CRKP ranged from 0.5 to 32 μg/mL and <0.5 to 32 μg/mL, respectively. MIC_50_ and MIC_90_ values were identical (16 μg/mL) in 35 colistin-susceptible KPC-2-producers. Those same values were respectively 16 and 32 μg/mL in 44 colistin-susceptible non-KPC-2-producers, 4 and 16 μg/mL in 25 colistin-resistant KPC-2-producers, and 16 and 32 μg/mL in 30 colistin-resistant non-KPC-2-producers.

### 2.2. AS101 Antibacterial Activity Characterization 

**Figure 1:**
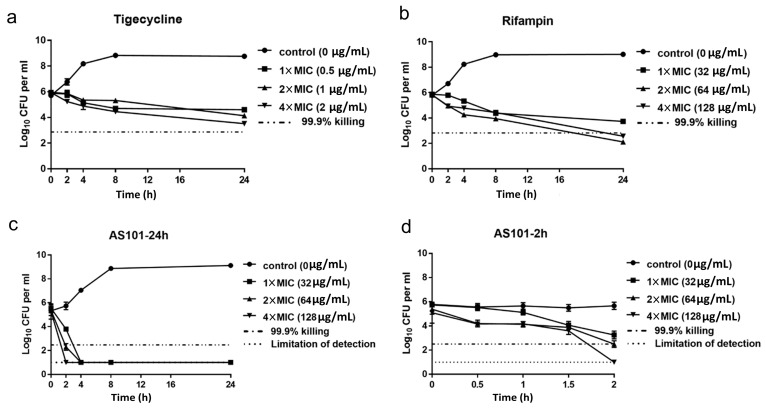
Time-kill kinetic assays of AS101 against carbapenem-resistant *K. pneumoniae* ATCC BAA-1705. Following treatment with 1× MIC (filled squares), 2× MIC (filled triangles), or 4× MIC (filled inverted triangles) (Table S1) of (**a**) tigecycline, (**b**) rifampin, (**c**) AS101, or untreated (control, filled circle), carbapenem-resistant *K. pneumoniae* ATCC BAA-1705 viability was measured for 2, 4, 8 and 24 h. (**d**) Due to its effective antibacterial activity, short-term kinetic assays were performed for AS101. Points, means; bars, standard deviations for triplicate experiments. Dotted-dashed lines indicate a 99.9% reduction of beginning inoculum. Dotted lines indicate detection limits. CFU, colony-forming unit. See also Figure S4 for results of CRE-723.

To evaluate AS101 antibacterial activity, time-kill assays were performed using CRKP ATCC BAA1705 and the colistin-resistant CRKP isolate CRE-723 (Table S1). According to our results for tigecycline (bacteriostatic) and rifampin (bactericidal) (Figure 1a,b), AS101 demonstrated a faster bactericidal effect compared to rifampin, killing 99.9% of bacterial inoculum within 4 h (Figure 1c). Results from a short-term time-kill assay indicate 97.9%, 96.8%, and 96.8% bacterial eradication at 1×, 2×, and 4× MICs within 1.5 h, respectively, and >2-log_10_ (>99%) beginning inoculum reduction at all three treatment levels after 2 h (Figure 1d). Similar results were observed for CRE-723 (Figure S4): 98.8%, 99.8%, and 99.9% bacterial eradication at 1×, 2×, and 4× AS101 MICs within 1.5 h and >99.9% bacterial eradication within 2 h at all three concentrations. These findings confirm the status of AS101 as a quick-acting antibacterial agent. 

**Figure 2:**
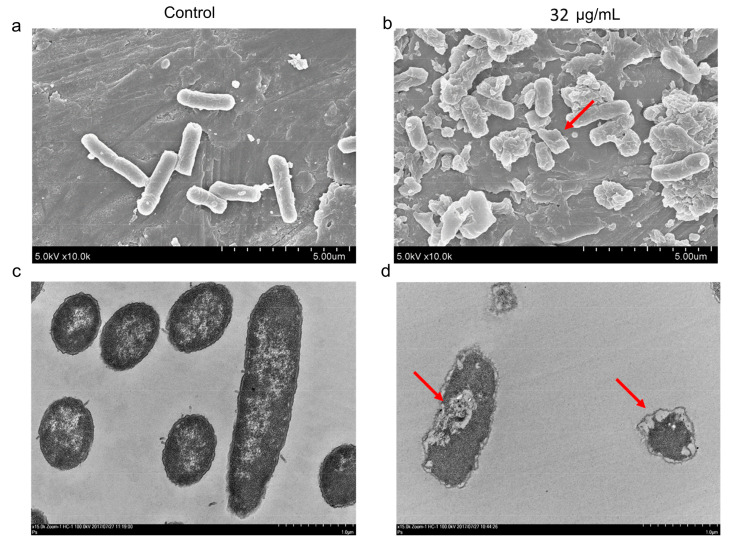
Electron microscopy images for AS101-treated *K. pneumoniae* ATCC BAA-1705. Scanning electron microscopy (SEM) and transmission electron microscopy (TEM) micrographs were respectively captured at 10,000× (**a**,**b**), and 15,000× magnification (**c**,**d**). Untreated (control) bacteria morphology remained intact and smooth (**a**,**c**). Leaking wrinkly surfaces (red arrows in (**b**,**d**)) were observed following exposure to 1× MIC (32 μg/mL) AS101. See also Figure S5 for results of CRE-723.

To further clarify the bactericidal effects of AS101, scanning, and transmission electron microscopy (SEM and TEM) were used to identify both internal and external morphological changes in the two isolates following treatment with 1× MIC AS101. SEM images indicate clear differences in membrane morphology between treated and untreated conditions in terms of leaking and wrinkly surfaces, as well as cell lysis (Figure 2a,b and Figure S5a,b). TEM micrographs show a corrugated outer layer and membrane perforations post-AS101 treatment (Figure 2c,d and Figure S5c,d). An empty cell envelope was also observed in the TEM micrographs (Figure S5d). Visual evidence indicates that AS101 altered bacterial cell permeability and perforated cell membranes, causing bacterial cell deformations.

### 2.3. AS101 Oxidative Damage Leads to Cell Death

Pharmacological manipulations of AS101 against *K. pneumoniae* isolates were performed with various chemical agents (Table S2). Compared to an untreated control, 320 mM of mannitol (a hydroxyl scavenger) increased MIC values 4-fold for both ATCC BAA-1705 and CRE-723—a strong indication that oxidative damage is an important antibacterial mechanism. Further, treatment with a divalent cation chelating agent (EDTA) altered outer membrane stability, enhanced AS101 permeability, and increased sensitivity to AS101 8-fold for ATCC BAA-1705 and 16-fold for CRE-723. According to our mechanistic analysis data, we hypothesize that AS101 could induce reactive oxygen species (ROS) pressure after penetrating outer membranes.

**Figure 3:**
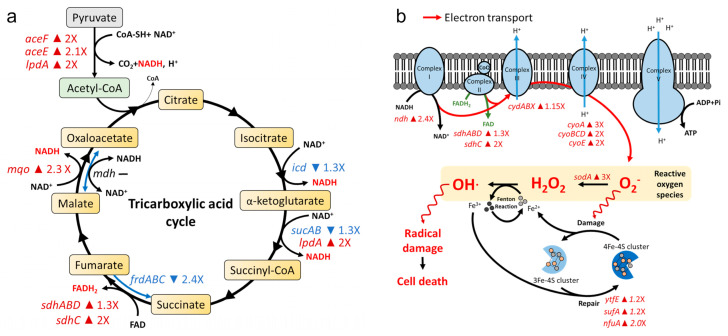

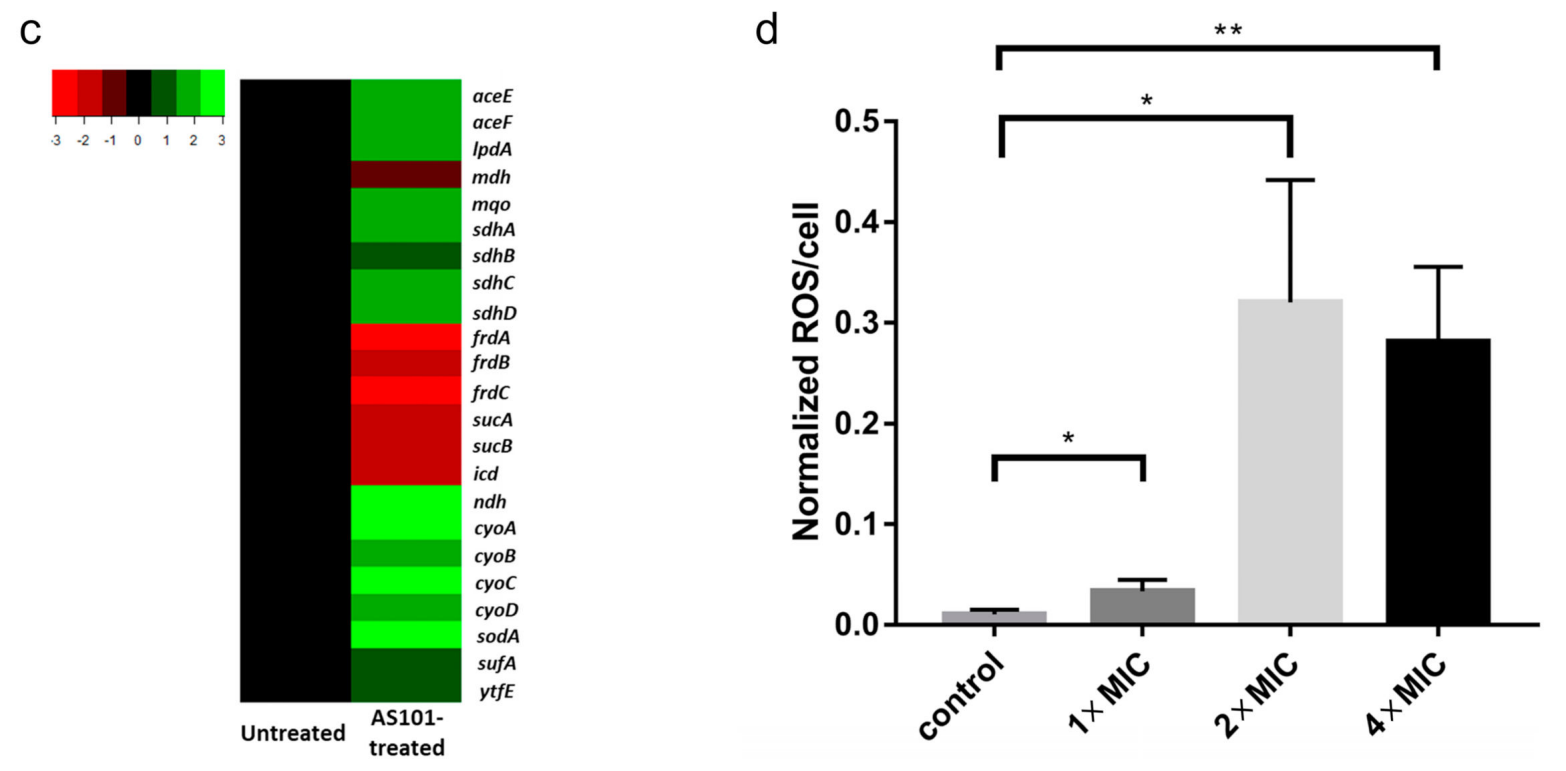
Illustration of the mechanistic analysis of AS101 against K. pneumoniae ATCC BAA-1705. According to the results in Table S2, gene expressions of (**a**) the tricarboxylic acid (TCA) cycle, and (**b**) oxidative phosphorylation on cell membranes were analyzed. Up-regulated genes with fold changes are shown in red, down-regulated genes in blue. A short horizontal line suggests no change in expression. Arrows indicate reaction direction. Boxes show reaction substrates, with byproducts next to each curved arrow. (**c**) Differentially expressed genes in (**a**,**b**) between the control and AS101-treated group. (**d**) ROS levels in K. pneumoniae ATCC BAA-1705 were determined following exposure to 1× MIC (32 μg/mL), 2× MIC (64 μg/mL), or 4× MIC (128 μg/mL) AS101, or 5% ethanol as a control (0 μg/mL). See also: Figure S6 for ROS levels of CRE-723. * *p* < 0.05; ** *p* < 0.01.

To clarify gene responses to AS101 treatment, we performed RNA microarrays to determine transcriptional profiles and identified over 150 differentially regulated genes (altered 2-fold or more). Results from gene ontology (GO) analyses indicate links between the highest upregulated genes and cellular respiration, electron transport chains, and oxidoreductase activity (Figure 3a,b). Following AS101 treatment, we observed an association between enhanced acetyl-CoA production and the upregulation of *aceEF* and *lpdA* genes, both of which encode a pyruvate dehydrogenase complex (Figure 3a,c). The up-regulation of succinate dehydrogenase (*sdhABCD*), dihydrolipoyl dehydrogenase (*lpdA*), and malate:quinone oxidoreductase (*mqo*) and the down-regulation of fumarate reductase (*frdABC*) and α-ketoglutarate dehydrogenase (*sucAB*) triggered increased tricarboxylic acid cycle (TCA cycle) activity, which in turn led to NADH and FADH_2_ accumulation (Figure 3a). Reducing equivalents promoted the expression of NADH dehydrogenase (cytochrome complex I encoded by *ndh*), succinate dehydrogenase (cytochrome complex II encoded by *sdhABCD*), cytochrome bd oxidase (cytochrome complex III encoded by *cydABX*), and cytochrome bo oxidase (cytochrome complex IV encoded by *cyoABCDE*) in electron transport chains, with electron leaks producing O_2_^−^ superoxide. O_2_^−^ accumulation led to superoxide-mediated iron–sulfur cluster destabilization and Fenton reaction stimulation, resulting in hydroxyl radical production, intracellular damage, and cell death [20].

ROS detection in *K. pneumoniae* ATCC BAA-1705 and CRE-723 at different AS101 concentrations confirmed our transcriptional analysis results (Figure 3d and Figure S6). Compared to the untreated control, significant increases in ROS levels were observed at 1× MIC (*p* < 0.05), 2× MIC (*p* < 0.05) and 4× MIC (*p* < 0.01) AS101. In summary, our data indicate that oxidative stress contributed to AS101 bactericidal effects against *K. pneumoniae*. 

### 2.4. In Vivo Antibacterial Activity of AS101 in Two Animal Models

**Figure 4:**
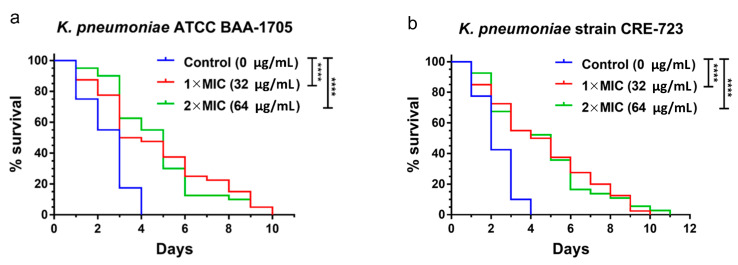
AS101-conferred treatment effects to *C. elegans* against *K. pneumoniae* infection. Nematodes (40 per group) were infected with (**a**) carbapenem-resistant *K. pneumoniae* (CRKP) ATCC BAA-1705, or (**b**) colistin-resistant CRKP isolate CRE-723, and treated with AS101 at various concentrations. Nematode survival was recorded daily. **** *p* < 0.0001.

Nematodes were infected with either CRKP ATCC BAA-1705 or CRE-723 prior to transfer to plates containing AS101 or a control. Compared to the untreated control group, AS101 resulted in a significant right-shift in the survival curve against the CRKP ATCC BAA-1705 strain in 1× and 2× MIC (both *p* < 0.0001) treatment groups (Figure 4a). Further, median nematode survival times were significantly extended to 3.5 days (*p* < 0.0001) at 1× MIC and 5 days (*p* < 0.0001) at 2× MIC (Table S3), with significant decreases in mortality risk observed at both 1× MIC (RR 0.32; 95% CI 0.19 to 0.54) and 2× MIC (RR 0.33; 95% CI 0.20 to 0.54). Significant AS101 treatment effects were also noted against colistin-resistant CRE-723—specifically, increased survival ratios were observed at 1× MIC (32 μg/mL, *p* < 0.0001) and 2× MIC (64 μg/mL, *p* < 0.0001) (Figure 4b), with median nematode survival times significantly extended to 4.5 days (*p* < 0.0001) at 1× MIC and 3.5 days (*p* < 0.0001) at 2× MIC (Table S3). Further, significant decreases in mortality risk were observed at 1× MIC (RR 0.35; 95% CI 0.21 to 0.58) and 2× MIC (RR 0.37; 95% CI 0.23 to 0.61). 

**Figure 5:**
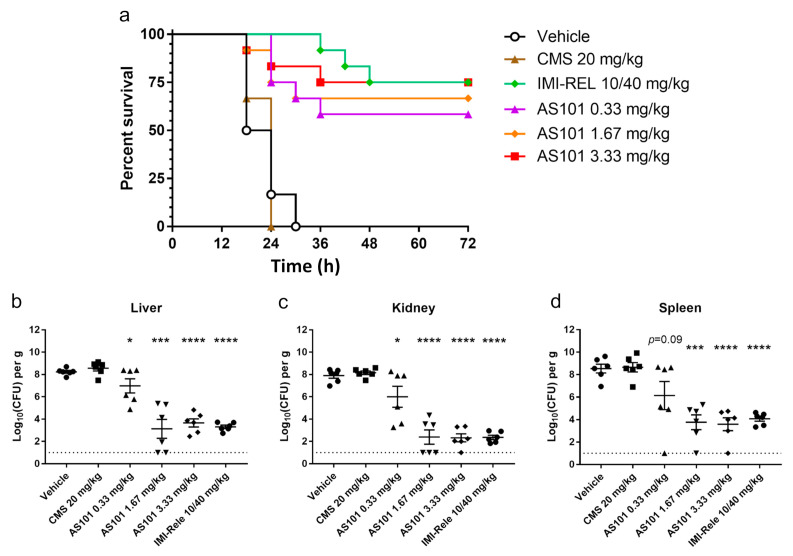
Lethally infected mice rescued by AS101 treatment. (**a**) CD-1 mice (*n* = 12 per group) were intraperitoneally injected with lethal challenges of the colistin-resistant CRKP isolate, CRE-723, and treated with 1× of a PBS vehicle, 20 mg/kg colistin methanesulfonate (CMS), 10/40 mg/kg imipenem-relebactam, or AS101 at various dosages. Infected mouse viabilities were observed as 0% (0/6) for the vehicle treatment; 0% (0/12) for 20 mg/kg CMS; 58.8% (7/12) for 0.33 mg/kg AS101; 66.6% (8/12) for 1.67 mg/kg AS101; 75% (9/12) for 3.33 mg/kg AS101; and 75% (9/12) for 10/40 mg/kg imipenem-relebactam. For organ load experiments, animals were intraperitoneally challenged with lethal doses of the colistin-resistant CRKP isolate, CRE-723, and euthanized 18 h post-infection. Bacterial counts in (**b**) livers, (**c**) kidneys, and (**d**) spleens were determined by plating. Dotted lines indicate detection limits. Data expressed as mean ± SD. * *p* < 0.05; *** *p* < 0.001; **** *p* < 0.0001.

CD1 mice were injected intraperitoneally with lethal doses of the colistin-resistant CRKP isolate CRE-723 to initiate systemic infections (Table S1). Infected mice were treated with imipenem-relebactam, colistin methanesulfonate (CMS), AS101, or a PBS vehicle at different concentrations. No differences between the CMS and vehicle groups were observed following lethal challenges with CRE-723 (Figure 5a), whereas 75% (9/12) of mice with imipenem-relebactam treatment survived. Daily treatments with AS101 at 0.33, 1.67 or 3.33 mg/kg dose-dependently improved infected mouse survival rates to 58.3% (7/12), 66.7% (8/12), and 75.0% (9/12), respectively.

To further evaluate AS101 treatment effects, livers, kidneys, and spleens were harvested from infected mice for bacterial load quantification. Similar bacterial loads were observed in the vehicle and CMS treatment group organs (Figure 5b–d). All mice with imipenem-relebactam were found with significantly decreased bacterial loads in livers, kidneys, and spleens. Significantly lower bacterial loads were found in the livers and kidneys (both *p* < 0.05) of mice treated with low dosages of AS101 (0.33 mg/kg), but not in spleens (*p* = 0.09). In comparison, significant reductions in bacteria levels were observed in the livers (*p* < 0.001), kidneys (*p* < 0.0001), and spleens (*p* < 0.001) of mice treated with the medium AS101 dosage (1.67 mg/kg). Significant decreases were also noted in organs collected from mice treated with the highest AS101 dosage (3.33 mg/kg, *p* < 0.0001 for all 3 organs).

## 3. Discussion

Antibiotic-resistant *K. pneumoniae* infections are considered a serious health issue worldwide, and the resistance to carbapenem and colistin is limiting treatment options [3,5,6,9]. The current drug development situation, in which few new antibiotics are being tested for use in clinical settings, is worsening the problem [21]. Ceftazidime-avibactam, a new β-lactam/β-lactamase inhibitor combination, was approved by the US Food and Drug Administration in 2015 for treating carbapenemase-producing (especially *K. pneumoniae* carbapenemase, or KPC), Gram-negative bacterial infections [22,23]. However, ceftazidime-avibactam resistance has already been identified in KPC-producing *K. pneumoniae* isolates, and many researchers believe that similar instances of antibiotic resistance will continue to emerge [24]. In another case, a chemically modified arylomycin named G0775 has been reported as demonstrating antibacterial activity against Gram-negative bacteria associated with multidrug-resistant *K. pneumoniae*, both in vitro and in vivo [25]. Time-consuming toxicological, pharmacological, and pharmacokinetic analyses are required before G0775 can be used for clinical applications. While researchers work on various solutions, drug repurposing is being promoted as a response to the financial and time requirements of drug development, and the number of studies involving existing preclinical profiles continues to grow [26]. We focused on the antibacterial agent AS101, for which phase II clinical trials are ongoing. Our results indicate that repurposed AS101 exhibits potent in vitro and in vivo effects in colistin- and carbapenem-resistant *K. pneumoniae* infections. A previous study reported that AS101 could indirectly rescue a cecal ligation-and-punctured mouse model via immunomodulation [27], and revealed activities against ESBL- and non-ESBL-producing *K. pneumoniae* (both at 10 μg/mL), as well as *Enterobacter cloacae* (9 μg/mL) [28,29]. These studies lend further support to the idea of AS101 as a potential antimicrobial agent. We found that AS101 exhibited potential antibacterial activity against CRKP isolates that are generally considered difficult to treat, within a MIC range of <0.5 to 32 μg/mL (Table 1). The results indicate antibacterial activity of AS101 against both colistin-susceptible and colistin-resistant CRKP isolates, with a maximum value (32 μg/mL) significantly lower than that measured for a 50% cytotoxic concentration of AS101 in Vero cells (145 μg/mL) [18]. We also observed AS101 effectiveness against colistin-resistant CRKP isolates both in vitro and in vivo. Adhering to animal welfare protection, an in vivo *C. elegans* model was used to evaluate the treatment effects. In the *C. elegans* infection model, our data indicate significant treatment effects from AS101 against *K. pneumoniae* (Figure 4). According to the treatment effects from AS101 that were found in a *C. elegans* infection model, the further evaluation was switched to a murine model with mono-bacterial sepsis. Daily treatments with AS101 dose-dependently and significantly improved infected mouse survival rates and the bacterial load on organs (Figure 5). Further, our data show that AS101 triggered increased TCA activity and ROS accumulation, similar to results reported for β-lactam, quinolone, and aminoglycoside bactericidal mechanisms [20], underscoring the potential use of AS101 for clinical purposes. However, AS101 was not fully approved by FDA, and thus, further clinical study of dosing range and distribution would be needed for its clinical usage (Appendix A).

## 4. Materials and Methods

### 4.1. Bacterial Strains

The 134 carbapenem-resistant *K. pneumoniae* (CRKP) isolates listed in this study were collected between 2012 and 2015 as part of a nationwide (Taiwan) surveillance study involving 16 hospitals [30]. All isolates were intermediate-resistant or resistant to at least one carbapenem antibiotic according to guidelines established by the Clinical and Laboratory Standards Institute (CLSI) [31]. Colistin resistance was defined as MIC > 2 μg/mL, as per guidelines from the European Committee on Antimicrobial Susceptibility Testing (EUCAST) [32].

### 4.2. Characterization of Bacterial Strains

Bacterial genotyping, including pulsotype, MLST, ESBL, and carbapenemase gene detection, and antimicrobial susceptibility testing for 134 CRKP isolates were performed as described in our previous studies [33,34].

### 4.3. Minimum Inhibitory Concentration (MIC)

AS101 antibacterial activity was determined by the broth microdilution method for 134 CRKP isolates and ATCC BAA-1705. Briefly, AS101 (Development Center for Biotechnology, Taipei, Taiwan) was dissolved in 99% ethanol (5% final concentration) and diluted with brain-heart infusion (BHI) broth (BD Difco™), followed by serial 2-fold dilution to 0.5–32 μg/mL per well. CRKP isolates were inoculated at a concentration of 5 × 10^5^ colony-forming units per ml (CFU/mL). A microplate reader was used to determine isolate growth after 16–20 h incubation at 37 °C [35]. Pharmacological manipulations were conducted by the addition of ethylenediaminetetraacetic acid (EDTA) (JT Baker) at 2 mM (outer membrane permeabilization), mannitol at 320 mM (hydroxyl scavenger), or calcium/magnesium ions at 10 mM (outer membrane charge).

### 4.4. Time-Kill Assays

To measure AS101 activity in vitro, time-kill assays were performed as described in a previous study [35]. Log-phase *K. pneumoniae,* ATCC BAA-1705 and CRE-723, were treated with AS101 in microplates at 1×, 2×, and 4× MIC, with 5% ethanol serving as a control. Serial 10-fold dilutions were created in 1×PBS at 0, 2, 4, 8, and 24 h and plated on LB agar (BD Difco™). Colonies were counted after 18 h incubation at 37 °C; 25–250 colonies per plate were used for detection purposes. Rifampin and tigecycline were used as bactericidal and bacteriostatic agent controls, respectively, when evaluating AS101 antimicrobial activity. Time-kill curves were constructed based on bacterial counts. Bactericidal activity was defined as a reduction < 3 log_10_ (99.9%) of total CFU/mL in the initial inoculum [35]. When analyzing AS101 bactericidal kinetics, serial 10-fold dilutions were created at 0, 0.5, 1.5, and 2.0 h to determine short-term treatment effects.

### 4.5. Electron Microscopy

Electron microscopy was used to observe the AS101 effects against *K. pneumoniae*. Bacterial cells were treated with AS101 at 32 μg/mL MIC for both CRKP ATCC BAA-1705 and colistin-resistant CRKP CRE-723 for 1 h prior to collection. Scanning electron microscopy procedures were performed as previously described [36]. For transmission electron microscopy, bacterial cells were treated and collected in the same manner and fixed, stained, and prepared as in an earlier study [37].

### 4.6. Gene Expression Profiling

Log-phase *K. pneumoniae* ATCC BAA-1705 bacterial suspensions were adjusted to a density of 5 × 10^5^ CFU/mL and treated with 1× MIC AS101 (5% ethanol used as control). As described in an earlier report [38], samples were collected at 20–30% inhibition compared to the control culture and preserved in Trizol^®^ reagent (Invitrogen) at −80 °C. RNA extraction and cDNA synthesis were performed as in our previous study [39]. Gene expression profiles were evaluated by RNA microarrays (Agilent) at the Welgene Biotech Company (Taipei, Taiwan) according to manufacturer protocols [40]. Genes with ≤0.05 false discovery rates and >2-fold changes in expression were selected for functional assays. An R package clusterProfiler was used for matching with GO and KEGG pathway databases [41].

### 4.7. Reactive Oxygen Species (ROS)

Cellular ROS levels were determined for *K. pneumoniae* ATCC BAA-1705 and CRE-723 using 2′,7′-dichlorodihydrofluorescein diacetate (DCFH-DA) oxidative stress assays [42]. After pre-incubating bacterial cells in BHI broth with 100 μM DCFH-DA (Sigma-Aldrich, St. Louis, MO, USA) for 2 h, treated cells were harvested and washed with 1× PBS, resuspended in BHI broth, and adjusted to appropriate densities. Cells were treated with AS101 on microplates for 1 h at 1×, 2×, or 4× MIC, with 5% ethanol used as a control. Fluorescent intensity was detected using spectrofluorometric readers at 500 and 530 nm wavelengths for excitation and emission, respectively. Results were normalized using viable bacterial counts in suspensions. All experiments were performed in triplicate. 

### 4.8. Nematode Survival Assays

*Caenorhabditis elegans* (strain N2) worms were maintained on nematode growth medium (NGM) agar plates with *E. coli* OP50 bacterial lawns at 20 °C. All procedures were executed as described in previous studies for the pulse-chase experiment [34,43]. Briefly, 300–400 growth-synchronized L4-stage worms were infected with *K. pneumoniae* ATCC BAA-1705 or the CRE-723 isolate. Next, 30–40 infected worms were transferred onto BHI agar with an *E. coli* OP50 bacterial lawn. AS101 was prepared in the agar at 1× or 2× MIC (5% ethanol as control) for each plate. Nematode survival was checked daily. To mimic daily treatment, live worms were transferred to new BHI agar plates under the same conditions. All assays were performed in triplicate.

### 4.9. Mouse Model 

Specific, pathogen-free (SPF) male ICR (CD1) mice aged 6–8 weeks were purchased from Lasco Biotechnology (Taipei, Taiwan) and held in SPF units in the Kaohsiung Medical University (KMU) Laboratory Animal Center for at least 1 week prior to use in all experiments. Procedures were submitted for approval by the KMU Institutional Animal Care and Use Committee (No. 106191; approved on 23 February 2018), and all animal experiments were conducted in accordance with KMU institutional guidelines. Mice were randomly placed into groups of 6 or 12, with numbers determined by empirical results from pilot experiments and according to statistical power requirements. To maximize blinding, all animal infection, treatment, and tissue processing procedures were performed by two independent researchers. All animal work was performed in an Association for Assessment and Accreditation of Laboratory Animal Care International (AAALAC)-accredited facility.

### 4.10. Bacterial Infection and Survival

The sepsis mouse infection model used in this research was established in accordance with previous studies [44,45]. Briefly, mice were injected intraperitoneally (i.p.) with lethal doses of *K. pneumoniae* isolate CRE-723 (1.5 × 10^8^ CFU). After 60 min, infected mice were treated with AS101, imipenem-relebactam (Merck), colistin (colistin methanesulfonate, CMS) (Santa Cruz), or a PBS vehicle. Daily AS101 injections were given at concentrations of 0.33, 1.67, or 3.33 mg/kg (approximately 10, 50, and 100 mg per mouse, respectively) [18]—far less than the 50% lethal dose (LD_50_) reported in an earlier in vivo toxicity study [19]. Imipenem-relebactam was i.p.-administered every 6 h at 10/40 mg/kg, with cilastatin also at 10 mg/kg, as described in a previous study [46]. CMS with a PBS vehicle was i.p. administered at 20 mg/kg four times per day as an invalid treatment [47]. Mouse survival was recorded every 6 h over 3 days.

### 4.11. Organ Bacterial Load

The same sepsis infection model was used to investigate organ bacterial load eradication following AS101 treatment. Mice were euthanized 16 h post-infection. Spleens, livers, and kidneys were collected, placed in 2 mL sterile PBS, and held on ice prior to homogenization. Homogenates were serially diluted in PBS, plated onto LB agar with 2 μg/mL colistin, and incubated at 37 °C for 16–18 h prior to CFU enumeration to quantify organ bacterial loads.

### 4.12. Statistical Analysis

Antibiotic susceptibility and gene detection profiles were visualized as ggplot2 heatmaps using RStudio (v.1.1.453). For time-kill assays, ROS detection, and organ bacterial load measurements, results were expressed as mean ± standard deviation and analyzed using Student’s *t*-tests. For survival tests, Kaplan–Meier curves were constructed with GraphPad Prism software (v.7.0) and analyzed using Mantel–Cox log-rank tests.

## 5. Conclusions

In summary, as an immunomodulation agent still undergoing phase II clinical trials, AS101 shows the potential for repurposing in response to the current need for new antibiotics. Our finding that AS101 exhibits antimicrobial activity against colistin-resistant and carbapenem-resistant bacteria encourages further research to examine that potential.

## Figures and Tables

**Table 1:** Antimicrobial susceptibility of AS101 against 134 CRKP isolates.

CRKP Isolate (n = 134)	KPC Producer ^1^	AS101 MIC (μg/mL)		
		MIC Range	MIC_50_	MIC_90_
Colistin-susceptible ^1^ (*n* = 79)	KPC-2 (+) (*n* = 35)	0.5–32	16	16
	KPC-2 (−) (*n* = 44)	1–32	16	32
Colistin-resistant ^1^ (*n* = 55)	KPC-2 (+) (*n* = 25)	<0.5–16	4	16
	KPC-2 (−) (*n* = 30)	1–32	16	32

Note: 32 μg/mL MIC against *K. pneumoniae* ATCC BAA-1705. ^1^ Detailed information was found in Figures S1–S3.

## Data Availability

Data is contained within the article and Supplementary Materials.

## Supplementary Materials

The following are available online at https://www.mdpi.com/article/10.3390/ph14080795/s1. Figure S1: Heatmap visualization of antimicrobial-resistant profiles for 134 carbapenem-resistant *K. pneumoniae* isolates. Figure S2: ESBL and carbapenemase gene distributions in 134 carbapenem-resistant *K. pneumoniae* isolates. Figure S3: Phylogenic tree for 134 carbapenem-resistant isolates. Figure S4: Time-kill kinetic assay data for AS101 against colistin- and carbapenem-resistant *K. pneumoniae* isolate CRE-723.Figure S5: Electron micrographs of colistin- and carbapenem-resistant *K. pneumoniae* isolate CRE-723 treated with AS101.Figure S6: ROS detection in AS101-treated colistin- and carbapenem-resistant *K. pneumoniae* isolate CRE-723.Table S1: MIC values of *K. pneumoniae* ATCC BAA-1705 and CRE-723 to agents tested in this study. Table S2: AS101 MIC values for pharmacological manipulations against *K. pneumoniae* ATCC BAA-1705 and CRE-723.Table S3: Treatment effect of AS101 in *C. elegans* model.
