# Supplementary material for: Evaluation of the Organotellurium Compound AS101 for Treating Colistin- and Carbapenem-Resistant Klebsiella pneumoniae
Source: Pharmaceuticals (Basel). 2021 Aug 12;14(8):795. doi: 10.3390/ph14080795 (PMC8400984; doi:10.3390/ph14080795)
Supplement: Supplementary file 1 [file pharmaceuticals-14-00795-s001.zip › pharmaceuticals-1313302-supplementary.pdf]

Supplementary Materials

Figure S1. Heatmap visualization of antimicrobial-resistant profiles for 134 carbapenem-resistant *K. pneumoniae* isolates. Antibiotic susceptibilities: light pink, susceptible; pink, intermediate; dark pink, resistant. Antibiotic-resistant profiles were determined for aztreonam (ATM; 132/134, 98.5%), ceftazidime (CAZ; 132/134, 98.5%), cefazolin (CFZ; 134/134, 100%), ciprofloxacin (CIP; 129/134, 96.3%), doripenem (DOR; 134/134, 100%), ertapenem (ETP; 133/134, 99.3%), cefepime (FEP; 128/134, 95.6%), cefoxitin (FOX; 133/134, 99.3%), cefotaxime (FTX; 134/134, 100%), ceftriaxone (FRX; 134/134, 100%), imipenem (IMP; 133/134, 99.3%), levofloxacin (LEV; 126/134, 94.0%), meropenem (MEM; 129/134, 96.3%), trimethoprim/sulfamethoxazole (SXT; 117/134, 87.3%), piperacillin-tazobactam (TZP; 133/134, 99.3%), colistin (COL; 55/134, 41.0%), gentamicin (GEN; 86/134, 64.2%), and amikacin (AMK; 43/134, 32.1%). Differences between isolate groups were categorized according to antibiotic-resistant profiles.

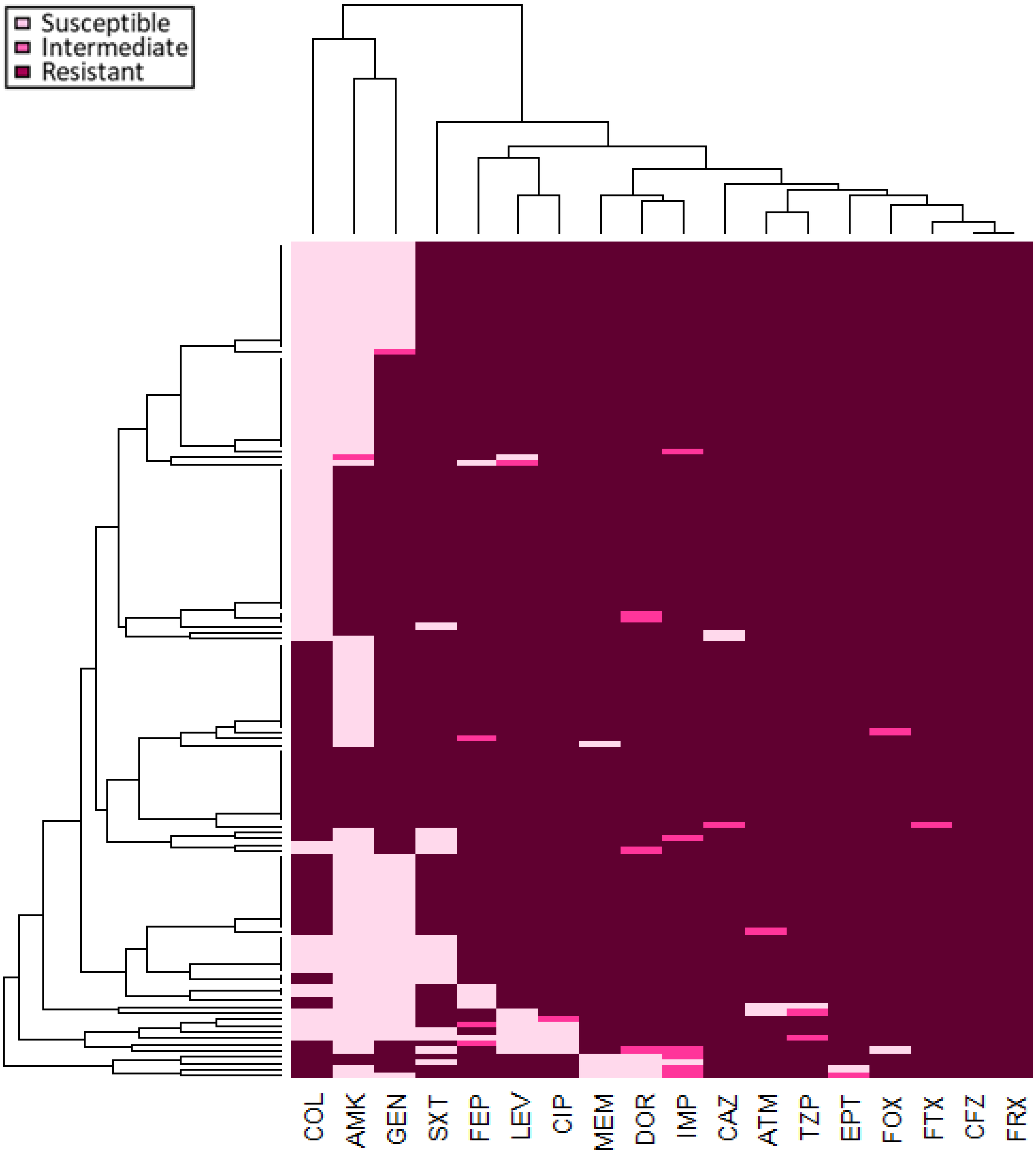

Figure S2. ESBL and carbapenemase gene distributions in 134 carbapenem-resistant *K. pneumoniae* isolates. Black and grey colors indicate positive and negative PCR detection results, respectively. Differences between isolate clusters were categorized according to gene carriages or distributions.

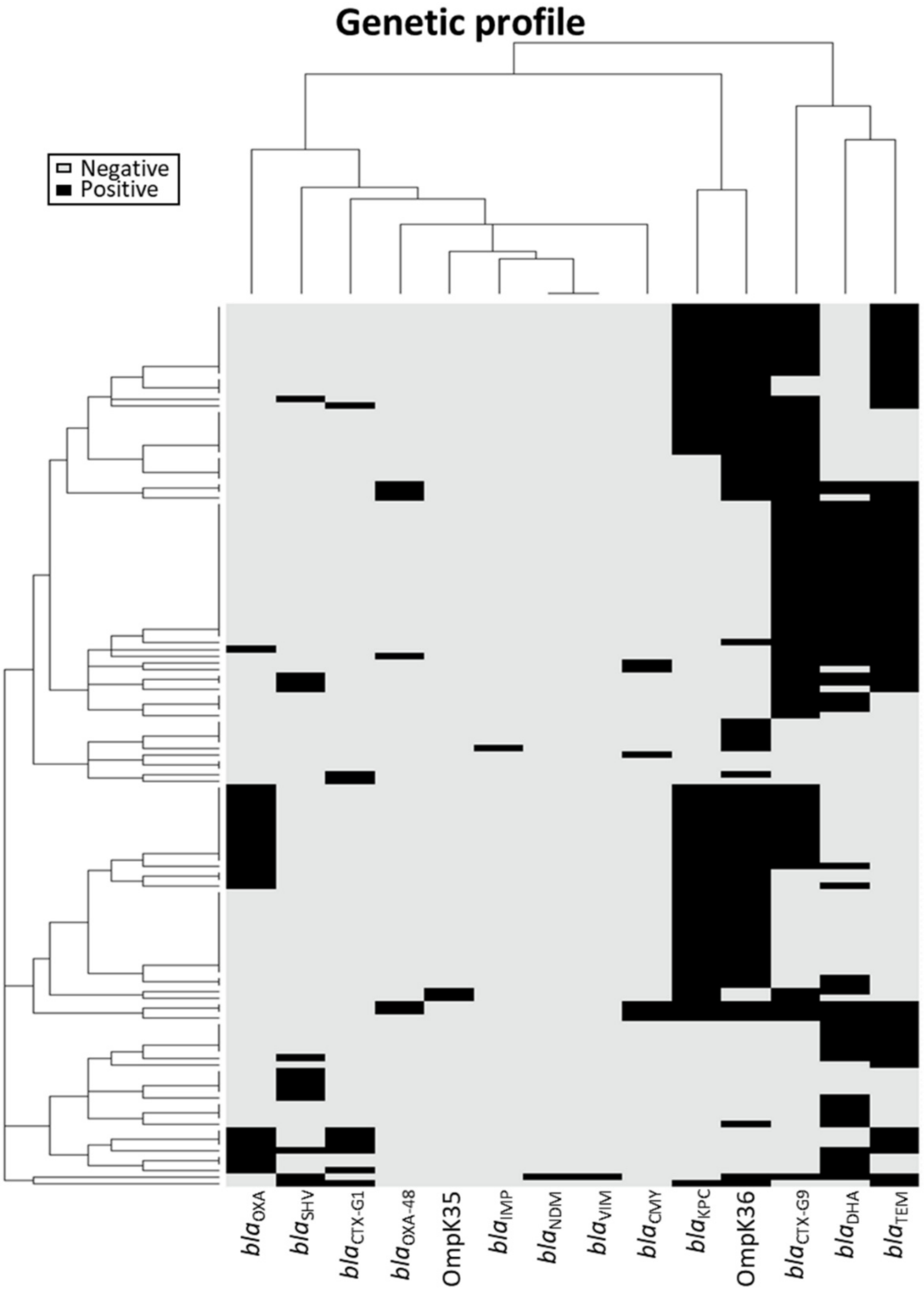

Figure S3. Phylogenic tree for 134 carbapenem-resistant isolates. A pulsotype relationship dendrogram was constructed according to Dice similarity and unweighted pair group method with arithmetic mean (UPGMA), using GelComparII software version 6.5 (Applied Maths, Sint-Martens-Latem, Belgium). Pulsotypes were assigned to the same cluster if they exhibited 70% similarity. Multi-locus sequence types are shown next to isolate codes.

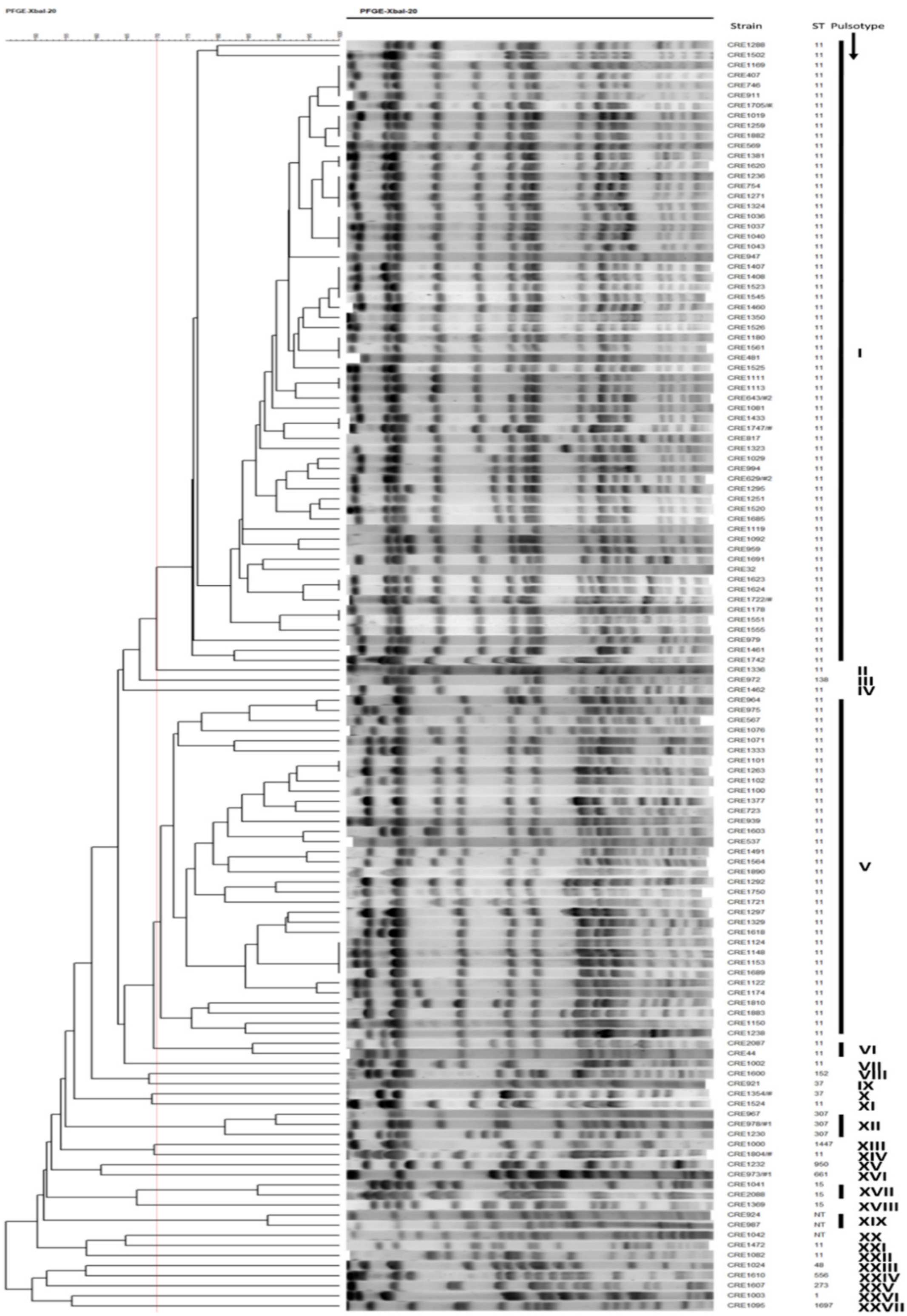

Figure S4. Time-kill kinetic assay data for AS101 against colistin- and carbapenem-resistant *K. pneumoniae* isolate CRE-723. Following treatment with 1x MIC (filled squares), 2x MIC (filled triangles), 4x MIC (filled inverted triangles) or untreated control (filled circles) of (**a**), tigecycline; (**b**), rifampin; or (**c**), AS101, colistin-resistant CRKP isolate CRE-723, viability was measured at 2, 4, 8 and 24 h. (**d**), Short-term assays were performed to create an AS101 antibacterial activity profile at 2 h. Data are expressed as mean±SD for triplicate experiments. Dashed-dotted lines indicate a 99.9% reduction of beginning inoculum; dotted lines indicate detection limits. CFU, colony-forming unit.

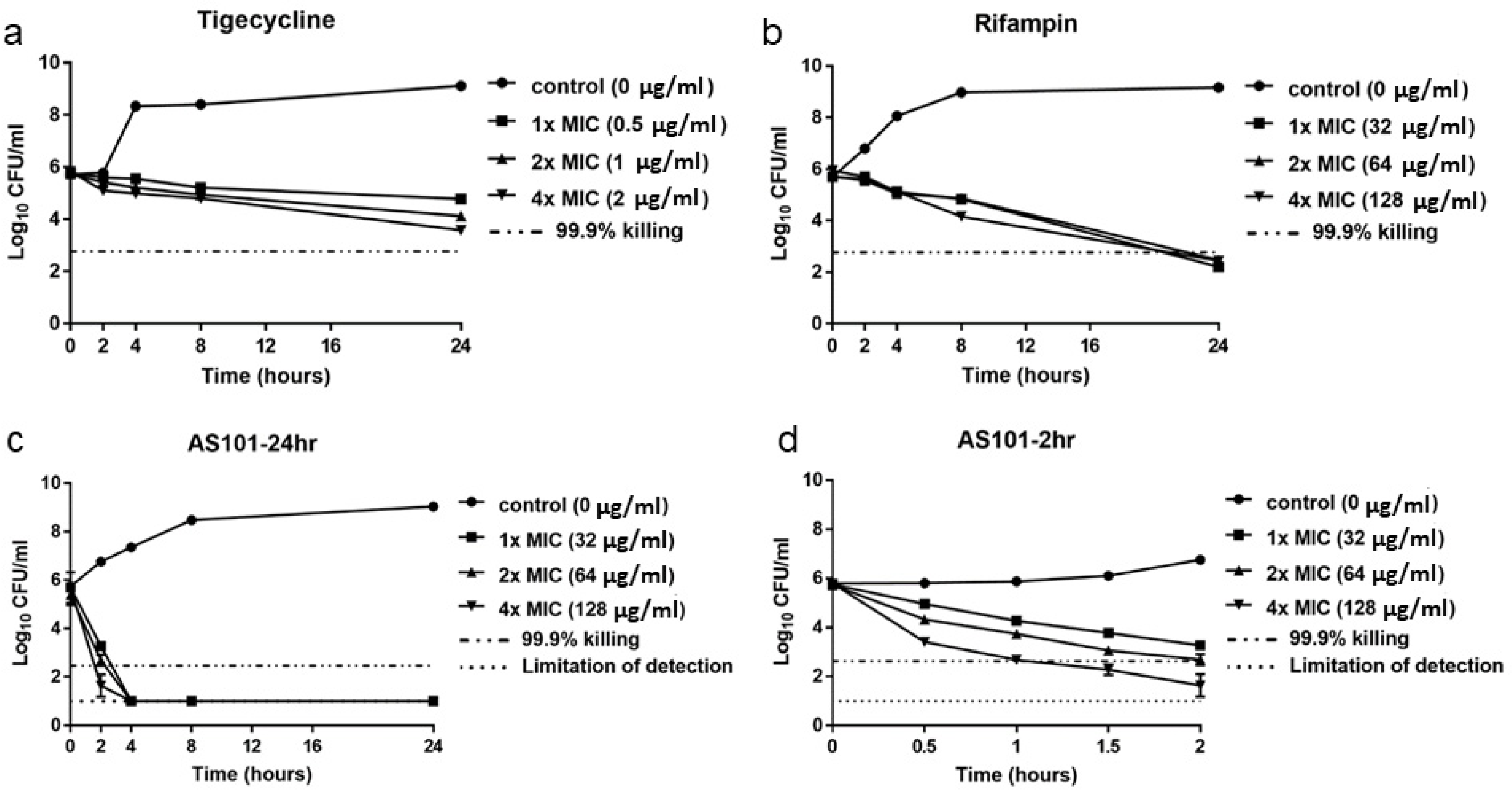

Figure S5. Electron micrographs of colistin- and carbapenem-resistant *K. pneumoniae* isolate CRE-723 treated with AS101. Scanning electron microscopy (SEM) and transmission electron microscopy (TEM) micrographs were captured at (**a**-**b**), 5,000x magnification and (**c-d)**, 100,000x magnification, respectively. (**a**) and (**c**), Untreated (control) bacterial morphologies remained intact and smooth. (**b**) and (**d**),) red arrows: Leaking and wrinkly surfaces and empty cell envelopes were observed following exposure to 1x MIC (32 μg/ml) AS101.

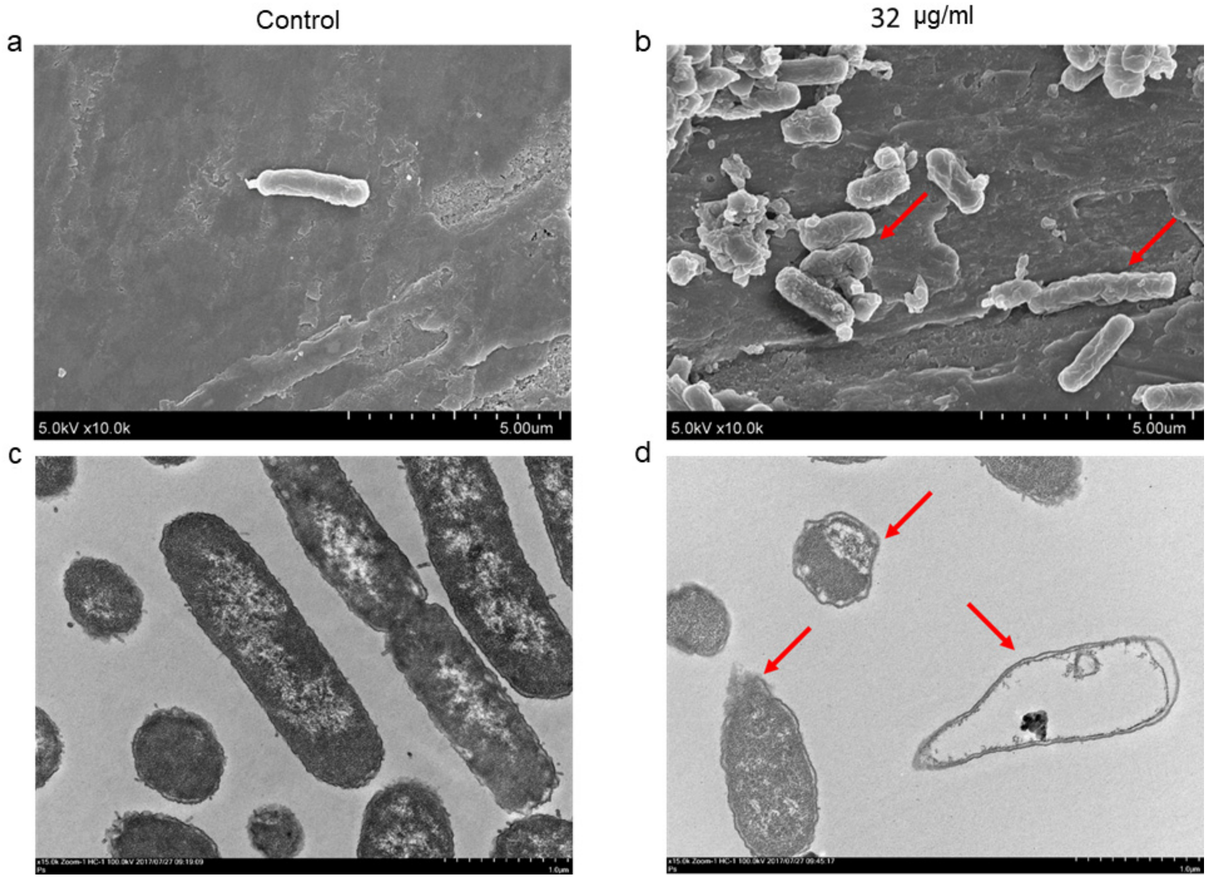

Figure S6. ROS detection in AS101-treated colistin- and carbapenem-resistant *K. pneumoniae* isolate CRE-723. ROS levels were determined following exposure to 1x MIC (32 µg/ml), 2x MIC (64 µg/ml), or 4x MIC (128 µg/ml) AS101, or 5% ethanol as a control (0 µg/ml). Data are normalized to viable bacterial counts and expressed as mean±SD. *, p<0.05; **, p<0.01.

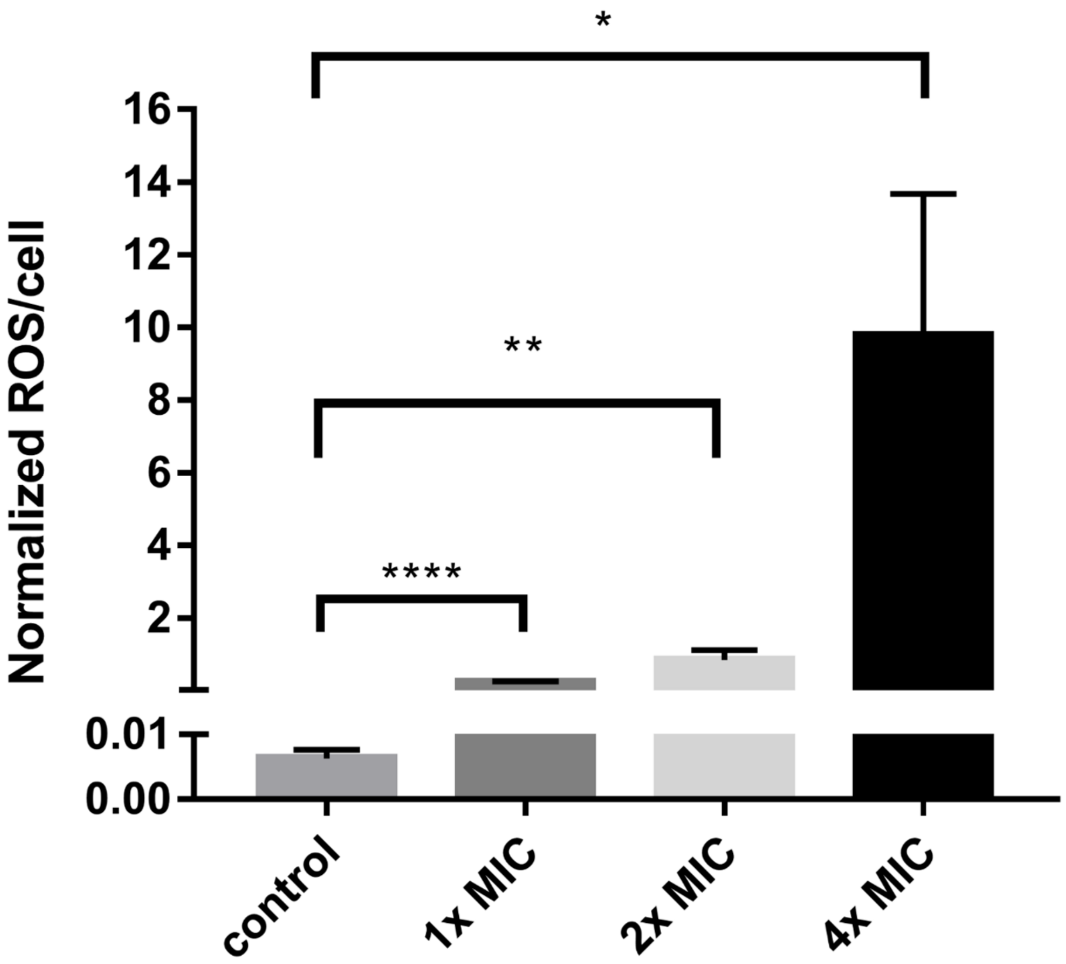

Table S1. MIC values of *K. pneumoniae* ATCC BAA-1705 and CRE-723 to agents tested in this study.

***K. pneumoniae* strain**	**MICs to antimicrobials (μg/ml)**					
	**AS101**	**Tigecycline**	**Rifampin**	**Colistin**	**Imipenem**	**Imipenem-Relebactam**
ATCC BAA-1705	32	0.5	32	0.5	4	0.125/4
CRE-723	32	0.5	32	>1024	8	0.25/4

Table S2. AS101 MIC values for pharmacological manipulations against *K. pneumoniae* ATCC BAA-1705 and CRE-723.

**Strain**	**AS101 MIC (µg/ml)**	
	K. pneumoniae **ATCC BAA-1705**	***K. pneumoniae*** **CRE-723 isolate**
Control	32	32
Mannitol 320 mM	128	128
EDTA 2 mM	4	2
Ca2+ 10 mM	32	32
Mg2+ 10 mM	32	32

Table S3. Treatment effect of AS101 in *C. elegans* model

Strain	Group	Survival (days)		p value	Risk ratio	95% CI	
		Mean ± SD	Median			Lower	Upper
K. pneumoniae ATCC BAA-1705	1x MIC	4.68 ± 0.44	3.5	< 0.0001	0.32	0.19	0.54
	2x MIC	4.73 ± 0.35	5		0.33	0.20	0.54
	control	2.45 ± 0.16	3		1.00	-	-
K. pneumoniae CRE-723	1x MIC	4.63 ± 0.44	4.5	< 0.0001	0.35	0.21	0.58
	2x MIC	4.35 ± 0.41	3.5		0.37	0.23	0.61
	control	2.3 ± 0.15	2		1.00	-	-
